# Distal Cholangiocarcinoma Diagnosed via Percutaneous SpyGlass Cholangioscopy After Failed Endoscopic Retrograde Cholangiopancreatography (ERCP) in a Patient With Suspected Gallstone Pancreatitis

**DOI:** 10.7759/cureus.105913

**Published:** 2026-03-26

**Authors:** Kian Memari, Sara Ali, Anastasiya Sizova, Emily Reinoso, Lissette P Lazo, Shane Williams, Peter Cohen

**Affiliations:** 1 Family Medicine, Palmetto General Hospital, Hialeah, USA; 2 Medicine, Nova Southeastern University Dr. Kiran C. Patel College of Osteopathic Medicine, Clearwater, USA; 3 Medicine, Nova Southeastern University Dr. Kiran C. Patel College of Osteopathic Medicine, Fort Lauderdale, USA; 4 Family Medicine, Nova Southeastern University Dr. Kiran C. Patel College of Osteopathic Medicine, Fort Lauderdale, USA

**Keywords:** biliary malignancy, cholangiocarcinoma, endoscopic ultrasound, ercp failure, obstructive jaundice, spyglass cholangioscopy

## Abstract

Cholangiocarcinoma is a rare and aggressive malignancy of the biliary epithelium that frequently presents with nonspecific symptoms and is often diagnosed at an advanced stage. Diagnosis may be delayed when coexisting hepatobiliary conditions obscure the underlying pathology, and conventional imaging or endoscopic retrograde cholangiopancreatography (ERCP) may fail to identify distal bile duct lesions, particularly when biliary access cannot be achieved.

We report the case of an 81-year-old man who presented with findings initially suggestive of gallstone pancreatitis but with evolving features concerning for persistent biliary obstruction. Imaging demonstrated pancreatitis with biliary dilation but no definitive obstructing lesion. ERCP was pursued due to persistent cholestatic liver enzyme abnormalities, common bile duct dilation, and concern for an underlying obstructive process; however, cannulation of the common bile duct was unsuccessful. Subsequent percutaneous transhepatic biliary drainage (PTBD) was performed to establish biliary access. Through this access, SpyGlass (Boston Scientific, Marlborough, MA, USA) single-operator cholangioscopy was advanced via the percutaneous tract, revealing an irregular distal bile duct stricture. Targeted biopsies confirmed distal cholangiocarcinoma.

The patient’s hospital course was complicated by *Escherichia coli* bacteremia secondary to biliary obstruction. Given poor surgical candidacy, internal biliary drainage was achieved via endoscopic ultrasound-guided choledochoduodenostomy with placement of a lumen-apposing metal stent. Despite appropriate biliary decompression, the diagnosis represented advanced malignancy requiring palliative management.

This case highlights the importance of maintaining suspicion for malignant biliary obstruction in atypical presentations of pancreatitis and demonstrates the role of alternative cholangioscopy access routes in establishing diagnosis following failed ERCP.

## Introduction

Cholangiocarcinoma is a malignant neoplasm arising from the epithelial lining of the bile ducts and may occur in intrahepatic, perihilar, or distal extrahepatic locations. Although relatively uncommon, its incidence has increased globally, particularly among older adults, and prognosis remains poor due to delayed diagnosis and limited curative options [1,2]. Advanced disease is frequently present at the time of diagnosis, with five-year survival rates remaining low.

Typical clinical manifestations include painless jaundice, pruritus, pale stools, dark urine, and cholestatic liver enzyme abnormalities. However, clinical presentation may be atypical when coexisting hepatobiliary conditions such as gallstone disease or pancreatitis obscure the underlying pathology [3,4]. In such scenarios, biliary dilation without a clearly identifiable obstructing lesion should prompt further evaluation for occult malignancy.

Endoscopic retrograde cholangiopancreatography remains a cornerstone modality for both diagnosis and therapeutic intervention in biliary obstruction. However, ERCP has inherent technical limitations, including failure of biliary cannulation, which may prevent definitive diagnosis and management [5]. Advances in endoscopic technology, including SpyGlass (Boston Scientific, Marlborough, MA, USA) single-operator cholangioscopy, have improved direct visualization and biopsy of biliary strictures, significantly enhancing diagnostic accuracy in indeterminate cases [6]. Furthermore, alternative access routes such as percutaneous transhepatic approaches allow for cholangioscopy when conventional endoscopic access is not feasible.

## Case presentation

An 81-year-old man with a history of atrial fibrillation on rivaroxaban, type 2 diabetes mellitus, hypertension, prior nephrectomy, and benign prostatic hyperplasia presented with a two-month history of intermittent fever, epigastric discomfort, nausea, and vomiting. The patient reported persistent symptoms with progressive intolerance to oral intake.

On presentation, he was hemodynamically stable. Physical examination was notable for mild epigastric tenderness without guarding or rebound. No overt jaundice was appreciated.

Laboratory evaluation revealed a cholestatic pattern of liver injury with elevated alkaline phosphatase (664 U/L), total bilirubin of 2.0 mg/dL, elevated transaminases, and markedly elevated lipase (1781 U/L). White blood cell count was within normal limits, and serum lactate was normal. These findings were consistent with acute pancreatitis in the setting of possible biliary pathology (Table 1).

**Table 1:** Detailed laboratory investigations µL: microliter; mm³: cubic millimeter; gm/dL: grams per deciliter; fL: femtoliter; pg: picogram; mmol/L: millimoles per liter; mg/dL: milligrams per deciliter; ng/mL: nanograms per milliliter; mIU/mL: milli-international units per milliliter; U/L: units per liter; mg/L: milligrams per liter; mL/min/1.73 m²: milliliters per minute per 1.73 square meters of body surface area; WNL: within normal limits; AST: aspartate aminotransferase; ALT: alanine aminotransferase

Parameter (unit)	Patient’s value	Normal reference range	Clinical note/interpretation
White blood cell (µL)	8.6	5.0 - 11.0 x 10^3^	Within normal limits (WNL)
Red blood cell (mm^3^)	4.23	4.70 - 6.10 x 10^6^	-
Hemoglobin (gm/dL)	13.0	14.0 - 18.0	-
Hematocrit (%)	38.2	42.0 - 52.0	-
Mean corpuscular volume (fL)	90	80 - 94	WNL
Mean corpuscular hemoglobin (pg)	30.6	27.0 - 31.0	WNL
Mean corpuscular hemoglobin concentration (gm/dL)	34.0	33.0 - 37.0	WNL
Red cell distribution width - coefficient of variation (%)	12.9	11.5 - 14.5	WNL
Platelet count (µL)	314	130 - 140 x 10^3^	WNL
Sodium (mmol/L)	134	137 - 145	-
Potassium (mmol/L)	3.7	3.4 - 5.0	WNL
Chloride (mmol/L)	103	98 - 107	WNL
Carbon dioxide (mmol/L)	25	22 - 30	WNL
Anion gap (mmol/L)	7	10 - 20	-
Blood urea nitrogen (mg/dL)	17	9.0 - 20.0	WNL
Creatinine (mg/dL)	0.70	0.66 - 1.25	WNL
Estimated glomerular filtration rate (mL/min/1.73 m^2^)	93	≥ 90	WNL
Glucose (mg/dL)	107	74.0 - 106.0	-
Lactic acid (mmol/L)	0.9	0.7 - 2.0	WNL
Calcium (mg/dL)	8.5	8.4 - 10.2	WNL
Total bilirubin (mg/dL)	2.00	0.20 - 1.30	-
AST (U/L)	268	17 - 59	-
ALT (U/L)	391	21 - 72	-
Alkaline phosphatase (U/L)	664	38 - 126	-
Lipase (U/L)	1781	23 - 300	-

**Figure 1:**
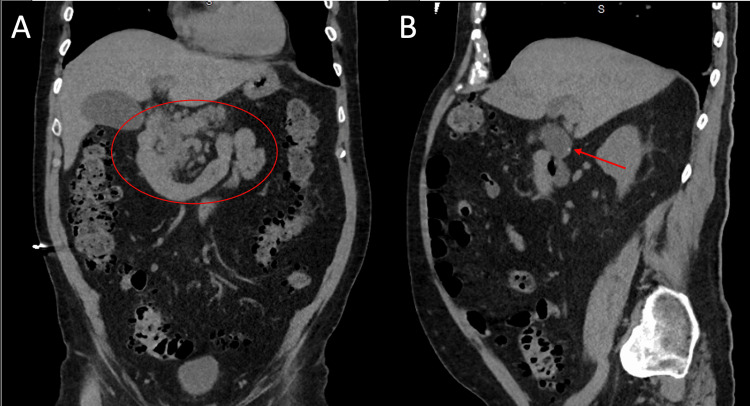
Contrast-enhanced CT of the abdomen and pelvis demonstrating acute pancreatitis and cholelithiasis (A) Diffuse pancreatic edema with surrounding peripancreatic fat stranding consistent with acute pancreatitis (red circle). (B) A 4-mm gallstone within the gallbladder lumen without imaging features of acute cholecystitis (red arrow).

Contrast-enhanced computed tomography of the abdomen and pelvis demonstrated diffuse pancreatic edema with surrounding inflammatory changes consistent with acute pancreatitis, as well as cholelithiasis without evidence of acute cholecystitis (Figure 1).

**Figure 2:**
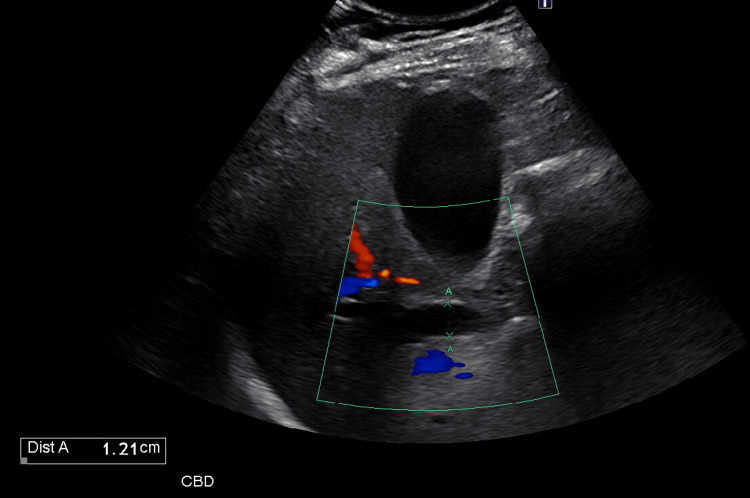
Right upper quadrant ultrasound demonstrating common bile duct dilation Color Doppler ultrasound image demonstrating dilation of the common bile duct measuring approximately 1.2 cm (light blue “X” measurement markers) within the region of interest outlined by the green box. No gallstones are visualized.

Right upper quadrant ultrasound confirmed dilation of the common bile duct measuring approximately 1.2 cm without visualization of choledocholithiasis (Figure 2).

**Figure 3:**
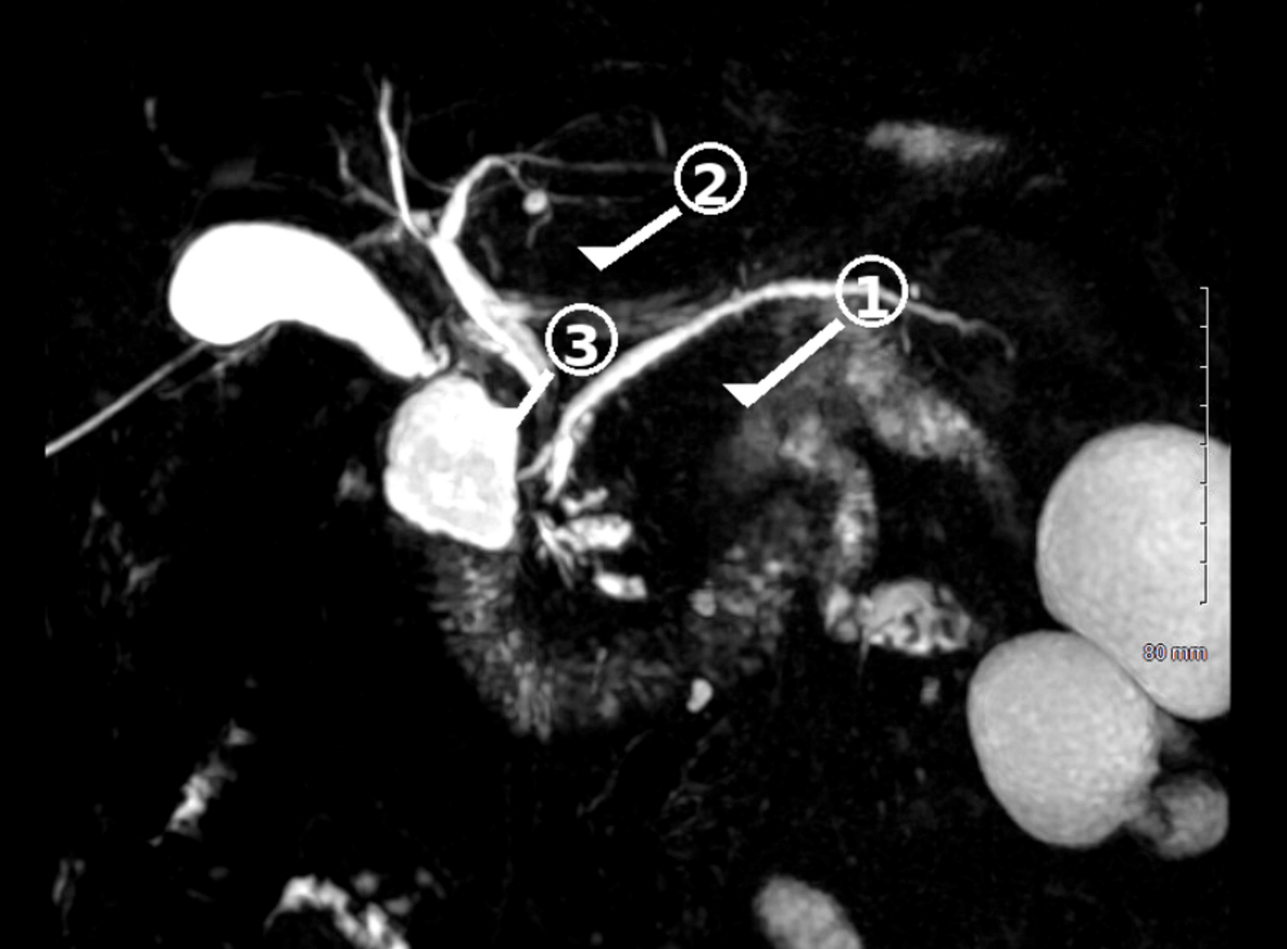
MRCP demonstrating pancreaticobiliary abnormalities Magnetic resonance cholangiopancreatography (MRCP), demonstrating (1) acute edematous interstitial pancreatitis involving the pancreatic tail, seen as mild pancreatic tail enlargement with surrounding inflammatory signal changes; (2) mild dilation of the main pancreatic duct; and (3) smooth tapering of the distal common bile duct (CBD) consistent with distal CBD stricture. These findings are associated with central intrahepatic and extrahepatic biliary dilation without evidence of choledocholithiasis.

Magnetic resonance cholangiopancreatography demonstrated edematous interstitial pancreatitis, mild dilation of the pancreatic duct, and smooth tapering of the distal common bile duct suggestive of a stricture, without evidence of an obstructing stone (Figure 3).

Although gallstone pancreatitis was initially suspected, several features raised concern for persistent biliary obstruction rather than transient stone passage. These included prolonged symptom duration over two months, persistent cholestatic liver enzyme abnormalities, common bile duct dilation without a visualized stone, and magnetic resonance cholangiopancreatography (MRCP) findings suggestive of distal ductal tapering. Taken together, these findings prompted further evaluation with ERCP.

ERCP was attempted but was unsuccessful due to the inability to cannulate the common bile duct. Given continued concern for biliary obstruction, percutaneous transhepatic biliary drainage was performed to achieve decompression and establish access to the biliary system.

**Figure 4:**
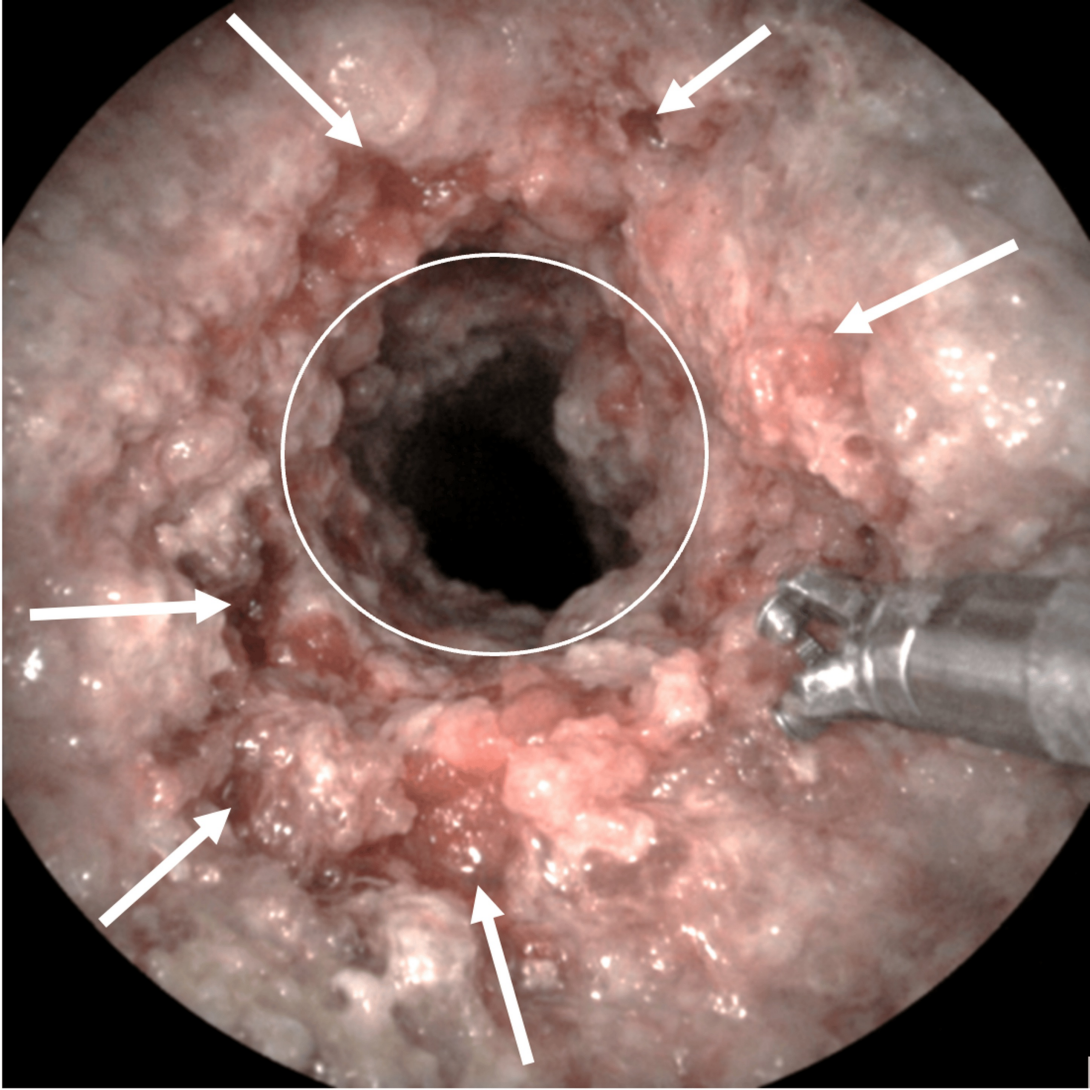
Distal common bile duct malignant stricture visualized via SpyGlass (Boston Scientific, Marlborough, MA, USA) cholangioscopy This image demonstrates an irregular, narrowed distal common bile duct (white circle) with friable mucosa and luminal distortion consistent with malignant obstruction (white arrows).

Through the established percutaneous tract, SpyGlass single-operator cholangioscopy was advanced, revealing an irregular, narrowed distal common bile duct with friable mucosa consistent with a malignant stricture. Direct visualization allowed for targeted biopsies of the lesion. A representative cholangioscopic image (Figure 4) demonstrates the irregular distal bile duct mucosa and luminal narrowing characteristic of malignant obstruction.

Histopathologic examination of biopsy specimens demonstrated moderately to poorly differentiated adenocarcinoma with a prominent desmoplastic stromal reaction, consistent with distal cholangiocarcinoma, with areas suspicious for lymphovascular invasion.

**Figure 5:**
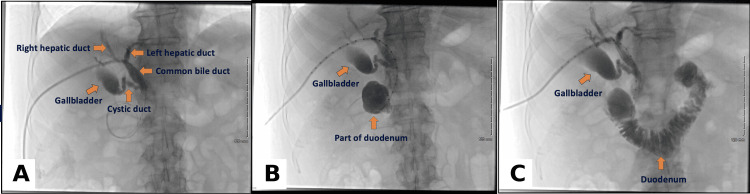
Fluoroscopic cholangiogram demonstrating biliary decompression (A) Fluoroscopic cholangiogram demonstrating biliary decompression. Arrows identify the right hepatic duct, left hepatic duct, common bile duct, cystic duct, and gallbladder. (B) Arrows highlight the gallbladder and a portion of the duodenum, demonstrating progression of contrast through the biliary system. (C) Imaging shows a decompressed biliary tree with contrast and bile flowing through the common bile duct stent into the duodenum, with arrows indicating the gallbladder and duodenum.

The hospital course was complicated by *Escherichia coli* bacteremia, attributed to biliary obstruction. The patient was treated with intravenous ceftriaxone with resolution of bacteremia. Given poor surgical candidacy, the external biliary drainage system was subsequently internalized via endoscopic ultrasound-guided choledochoduodenostomy with placement of a lumen-apposing metal stent. Post-procedural imaging confirmed adequate biliary decompression and improved bile flow into the duodenum (Figure 5).

## Discussion

This case illustrates a diagnostically challenging presentation of distal cholangiocarcinoma initially presumed to be gallstone pancreatitis. The overlap between benign biliary disease and malignant obstruction represents a well-recognized diagnostic pitfall, particularly in elderly patients.

In this case, the initial diagnosis of gallstone pancreatitis was supported by elevated lipase and the presence of cholelithiasis. However, several features were atypical for transient biliary obstruction, including prolonged symptom duration, persistent cholestatic liver enzyme abnormalities, and continued biliary dilation in the absence of a visible obstructing stone. Additionally, MRCP findings of smooth distal common bile duct tapering raised suspicion for an underlying stricture.

Although transient passage of a gallstone may produce temporary biliary dilation, such dilation typically resolves following relief of obstruction. In contrast, persistent ductal dilation and cholestatic biochemical abnormalities suggest ongoing obstruction, warranting further investigation. These considerations justified the decision to proceed with ERCP despite the presence of pancreatitis.

Failure of ERCP cannulation is a recognized limitation and may occur due to anatomic or technical factors. In such cases, percutaneous transhepatic biliary drainage provides an alternative route for decompression and access to the biliary system. The use of percutaneous SpyGlass cholangioscopy in this case represents a key diagnostic step, allowing direct visualization of the biliary mucosa and enabling targeted biopsy.

SpyGlass cholangioscopy has been shown to improve diagnostic yield in indeterminate biliary strictures compared with conventional brush cytology alone [6,7]. This is particularly important in distal cholangiocarcinoma, which may present as subtle or infiltrative lesions that are not readily detected on cross-sectional imaging.

The patient’s bacteremia highlights the increased risk of infection in the setting of biliary obstruction and underscores the importance of timely biliary decompression. The subsequent use of endoscopic ultrasound-guided choledochoduodenostomy allowed internalization of biliary drainage, providing a durable and less burdensome alternative to long-term external drainage.

This case emphasizes the importance of maintaining a high index of suspicion for malignancy in patients with atypical presentations of pancreatitis, particularly when clinical, laboratory, and imaging findings are not fully explained by benign etiologies.

## Conclusions

Distal cholangiocarcinoma may present with clinical features mimicking gallstone pancreatitis, leading to potential delays in diagnosis. Persistent biliary dilation, cholestatic laboratory abnormalities, and absence of a visualized obstructing stone should prompt further evaluation for underlying malignancy.

When ERCP fails to achieve biliary access, alternative strategies such as percutaneous cholangioscopy provide valuable diagnostic and therapeutic options. Early recognition of atypical features and appropriate use of advanced endoscopic techniques are essential for timely diagnosis and management of biliary malignancies.
